# Sugar-Sweetened Beverage Intake Trends in US Adolescents and Their Association with Insulin Resistance-Related Parameters

**DOI:** 10.1155/2010/196476

**Published:** 2009-09-06

**Authors:** Andrew A. Bremer, Peggy Auinger, Robert S. Byrd

**Affiliations:** ^1^Department of Pediatrics, University of California Davis School of Medicine, Sacramento, CA 95817, USA; ^2^Department of Neurology, University of Rochester School of Medicine and Dentistry, Rochester, NY 14642, USA

## Abstract

The purpose of this study was to evaluate current sugar-sweetened beverage (SSB) consumption trends and their association with insulin resistance-related metabolic parameters and anthropometric measurements by performing a cross-sectional analysis of the NHANES data during the years 1988–1994 and 1999–2004. Main outcome measures included SSB consumption trends, a homeostasis model assessment of insulin resistance, blood pressure, waist circumference, body mass index, and fasting concentrations of total cholesterol, HDL-cholesterol, LDL-cholesterol, and triglycerides. Although overall SSB consumption has increased, our data suggest that this increase was primarily due to an increase in the amount of SSBs consumed by males in the high-SSB intake group alone. Multivariate linear regression analyses also showed that increased SSB consumption was independently associated with many adverse health parameters. Factors other than SSB consumption must therefore be contributing to the increasing prevalence of obesity and metabolic syndrome in the majority of US children.

## 1. Introduction

The increased consumption of sugar-sweetened beverages (SSBs) over the past two decades has been implicated in the increased incidence of obesity and metabolic syndrome (MetS), a group of conditions associated with insulin resistance, including hypertension, dyslipidemia, central adiposity, and impaired glucose metabolism [1–3]. Based on data from the 1994–1996 Continuing Survey of Food Intakes by Individuals [4], the mean sugar consumption in all foods and beverages by Americans in the early 1990s comprised ~16% of their total daily energy intake. However, sugar intake from SSBs alone, which currently represent the largest single caloric food source in the US [5], now approaches or exceeds 15% of the daily caloric intake in several population groups, including adolescents [6, 7]. 

As in the adult population, the prevalence of obesity and MetS in the US pediatric population is increasing [8–15]. Although the reasons for this are unknown, the increased consumption of SSBs has been postulated to be a contributing factor [16–18]. Experimental studies support the hypothesis that SSBs may increase energy intake and induce weight gain via their reduced satiety response, the promotion of a positive energy balance by liquid calories relative to isoenergetic solid calories, and their dysregulation of energy homeostasis [2, 19–22]. Although not all studies support an association between SSB consumption and obesity [23, 24], SSB intake has nonetheless been associated with increased body weight, increased fat mass, dyslipidemia, and blood pressure [2, 25–29]. Furthermore, the odds of a pediatric patient becoming obese—and therefore at risk for developing MetS—is reportedly increased by ~60% for each additional SSB serving per day [16]; thus, the observation that the average intake of SSBs in US children and adolescents is now estimated to be more than double the amount consumed in the 1970s [30–32] has tremendous public health implications.

In order to evaluate the current consumption trends of SSBs and the association of SSB intake with insulin resistance-associated metabolic parameters and anthropometric measurements in the US pediatric population, we reviewed the National Health and Nutrition Examination Survey (NHANES) and performed these analyses in each available time period. We report our findings using data from US adolescents aged 12–19 years from NHANES III (1988–1994), NHANES 1999-2000, NHANES 2001-2002, and NHANES 2003-2004.

## 2. Methods

### 2.1. Study Design and Population

The NHANES is conducted by the National Center for Health Statistics of the Centers for Disease Control and Prevention (CDC), and is designed to monitor the health and nutritional status of the US civilian, noninstitutionalized population. NHANES III covered the years 1988–1994, and can be divided into two phases (1988–1991 and 1991–1994). Since 1999, NHANES has been planned and conducted as continuous annual surveys, and data are released in 2-year periods. A nationally representative sample is selected every year using a stratified multistage probability cluster sample design [33]; oversampling Mexican Americans and black individuals, adolescents aged 12–19 years, persons aged 60 years and older, low-income white individuals, and pregnant women permit more precise estimates for these groups. This report is based on data from NHANES III, NHANES 1999-2000, NHANES 2001-2002, and NHANES 2003-2004, as these were the most recently available NHANES that had released all the data needed for the inclusion criteria, exclusion criteria, and outcome variables. The NHANES protocol was approved by the National Center for Health Statistics institutional review board (IRB), and written informed consent was obtained from all participants 18 years of age and older; for adolescents younger than 18 years of age, written informed assent was obtained in addition to parent or guardian consent. This study was approved by the IRB at the University of California, Davis.

### 2.2. Subjects

The NHANES protocol consists of a home interview performed by a trained interviewer, followed by a visit to an examination center, where participants undergo physical examinations, provide blood and urine samples, and complete additional questionnaires. The details of the participant examinations and laboratory assessments are available on the NHANES website (www.cdc.gov/nchs/nhanes.htm). For our study, only data from participants aged 12 to 19 years were analyzed; individuals were excluded from analyses if they had not fasted for at least 8 hours, were pregnant, and/or used steroids, blood glucose regulators, insulin, other anti-diabetic agents, growth hormone, or sex hormones.

### 2.3. Measurements

The NHANES III Dietary Data Collection system and the United States Department of Agriculture (USDA) Survey Nutrient Database (SND) were used for dietary intake data Coding [34]. NHANES 1999–2001 utilized the University of Texas Food Intake Analysis System along with the SND for 1999-2000 coding and the USDA Food and Nutrient Database for Dietary Studies (FNDDS) for 2001 coding [35]. Survey Net, a computer assisted food coding and data management system developed by the USDA, and the FNDDS were used for NHANES 2002–2004 data [36, 37].

Outcome variables included glucose levels, insulin levels, a homeostasis model assessment of insulin resistance (HOMA-IR), total cholesterol (TC) levels, high-density lipoprotein cholesterol (HDL-C) levels, low-density lipoprotein cholesterol (LDL-C) levels, triglyceride (TG) levels, the TG/HDL-C ratio, systolic blood pressure (SBP), diastolic blood pressure (DBP), waist circumference (WC), and body mass index (BMI; calculated as weight in kilograms divided by height in meters squared) percentile for age and sex (per the National Center for Health Statistics references) [38]. Since fasting glucose and fasting insulin levels were not collected from the subjects in NHANES III, HOMA-IR (fasting glucose (mM/L)×fasting insulin (mU/mL)/22.5) [39] values from individuals in these cohorts could not be calculated. The TG/HDL-C ratio was included as an outcome variable due to its use as a marker of cardiovascular risk [40]. Mean WC is presented as the least squares mean, controlling for age and sex.

### 2.4. Definitions

Sugar-sweetened beverage information was obtained through a 24-hour dietary recall interview. (In NHANES 2003-2004, the 24-hour recall was assessed on two separate days; the first day was an in-person interview comparable to the previous NHANES study periods' primary interview mode, whereas the second day was a telephone interview 3–10 days later. For consistency in the methodology of data collection among the study periods, only the first day of the NHANES 2003-2004 24-hour recall was used in our analyses.) Sugar-sweetened beverages were defined as caloric soft drinks, colas, sugar-sweetened fruit drinks, or other sugar-sweetened beverages; pure fruit juices and diet soft drinks were not included. Sugar-sweetened beverage intake in grams (g) for each reported beverage was divided by 250 g (a serving equivalent; approximately 8 ounces [oz] or a cup of beverage) and summed for each adolescent. In each NHANES analyzed, low SSB intake was defined as the lowest quintile (≤20th percentile) of the sum of the number of SSB serving equivalents a subject consumed per day; medium was defined as the 2nd–4th quintiles (>20th–<80th percentile); high was defined as the highest quintile (≥80th percentile). Units of SSB intake are defined as the number of SSB serving equivalents per day. Physical activity information was obtained during the interview questionnaire. The amount of physical activity performed per day was determined by the sum of (the mean number of times a subject did activity per day)×(the average duration of each time in minutes)×(the metabolic equivalent [MET] score) [41–43]. Energy intake information was also obtained from the interview questionnaire to determine the subject's caloric intake per day (in kilocalories).

### 2.5. Statistical Analysis

Statistical analyses were performed with SUDAAN, version 9.0 (Research Triangle Institute, Research Triangle Park, NC) using techniques appropriate for the complex NHANES survey design. All of the analyses used the NHANES-provided sampling weights that were calculated to take into account unequal probabilities of selection resulting from the sample design, nonresponse, and planned oversampling of selected subgroups, so that results are representative of the US community-dwelling population. Dietary variables were analyzed both as continuous variables and in quintiles to minimize the chance that a small number of extreme observations would have undue influence on the results. The data are presented as NHANES III (1988–1994) versus NHANES 1999–2004, both in their individual components (i.e., NHANES III, Phase I 1988–1991; NHANES III, Phase II 1991–1994; NHANES 1999-2000; NHANES 2001-2002; and NHANES 2003-2004) as well as in their entirety (NHANES 1988–1994 and NHANES 1999–2004) since both trend and aggregate analyses were performed. Descriptive statistics summarize the data and are expressed as the mean ± the standard error (SE). Mean differences in outcomes comparing 1988–1994 to 1999–2004 in aggregate were tested for significance using *t*-tests. To test for linear trends, an ordinal variable representing the 5 time periods was included as a continuous dependent variable in regression analyses; each phase of NHANES III and each of the subsequent 2 year NHANES surveys were weighted to represent the US population [44]. Multivariate linear regression analyses were performed to determine independent associations between each outcome variable and the number of serving equivalents of SSBs consumed per day after adjusting for the amount of physical activity performed per day, age, sex, race, and energy intake per day (in kilocalories). All *P* values are 2-sided and statistical significance was established a priori at *α* = .05.

## 3. Results

### 3.1. Characteristics

The characteristics of the study participants are shown in Table 1. For NHANES III (1988–1994), a total of 3234 adolescents were studied; for NHANES 1999–2004, a total of 6967 adolescents were studied. The mean age, male/female ratio, and ethnic distributions between the study cohorts were comparable.

### 3.2. Trends in SSB Intake and Subgroup Analyses

The overall number of SSB serving equivalents consumed per day by each NHANES study period as well as the number of SSB serving equivalents consumed per day by the low-, medium-, and high-SSB intake groups from each cohort is shown in Table 2. A significant increase in overall SSB consumption over the 16 year time period was observed (*P* = .04 for trend analysis; *P* = .04 for aggregate analysis). However, in subgroup analyses, no significant differences were observed in the amount of SSB consumption in the low- and medium-SSB intake populations among the study cohorts. Although no significant difference was noted in the trends of SSB consumption in the high-SSB intake populations among the study cohorts, a significant difference was observed in the high-SSB intake groups when the NHANES III versus NHANES 1999–2004 data were analyzed in aggregate (*P* = .01). The high-SSB intake group in NHANES III consumed a mean of 6.6 SSB serving equivalents (~53 oz) per day, whereas the high-SSB intake group in NHANES 1999–2004 consumed a mean of 7.1 SSB serving equivalents (~57 oz) per day. For comparison, overall mean SSB consumption for the entire NHANES III and NHANES 1999–2004 cohorts were 2.8 SSB serving equivalents (~22 oz) per day and 3.0 SSB serving equivalents (~24 oz) per day, respectively. Thus, in each cohort, the high-SSB intake group was consuming over twice the overall mean number of SSB serving equivalents per day.

### 3.3. Sex-Specific Subgroup Analyses

Since the number of SSBs consumed per day may be different between males and females, we also performed sex-specific subgroup analyses; these results are shown in Table 3. In males, the pattern of overall SSB consumption trends and the significant differences observed between the high-SSB intake groups of NHANES III versus NHANES 1999–2004 mirror those found when studying the entire cohort. Specifically, a significant increase in male overall SSB consumption trends over the 16 year time period was observed (*P* = .048 for trend analysis); although the differences in male overall SSB consumption between NHANES III versus NHANES 1999–2004 did not reach statistical significance when the data were analyzed in aggregate, the analysis suggested a trend towards significance (*P* = .051). Moreover, as in the overall cohort analyses, a significant difference was observed in the male high-SSB intake groups when the NHANES III versus NHANES 1999–2004 data were analyzed in aggregate (*P* = .03). The male high-SSB intake group in NHANES III consumed a mean of 6.8 SSB serving equivalents (~54 oz) per day, whereas the male high-SSB intake group in NHANES 1999–2004 consumed a mean of 7.3 SSB serving equivalents (~58 oz) per day. For comparison, overall mean male SSB consumption for the NHANES III and NHANES 1999–2004 cohorts were 3.3 SSB serving equivalents (~26 oz) per day and 3.6 SSB serving equivalents (~29 oz) per day, respectively. Thus, overall mean SSB consumption was higher in males than in the overall cohort, especially in the high-SSB intake groups. In females, however, no significant changes were observed in either overall SSB consumption or the amount of SSB consumption in the low-, medium-, and high-SSB intake groups among the study cohorts. Moreover, SSB consumption by females (both overall and in each subgroup) in each study period was consistently less than that observed for the entire cohort.

### 3.4. Metabolic Parameters and Anthropometric Measurements Associated with SSB Intake

The multivariate linear regression analyses evaluating the relationship between SSB intake and insulin resistance-associated metabolic parameters and anthropometric measurements are shown in Table 4. All analyses were adjusted for the amount of physical activity performed per day, age, sex, race, and energy intake per day (in kilocalories). In the NHANES III cohort, each additional SSB serving equivalent consumed per day was associated with a 0.42 mg/dL decrease in HDL-C concentrations; HOMA-IR could not be assessed since fasting glucose and insulin concentrations were not obtained in the NHANES III study cohort. In the NHANES 1999–2004 cohort, each additional SSB serving equivalent consumed per day was associated with a 6% increase in HOMA-IR, a 0.16 mmHg increase in SBP, a 0.47 cm increase in WC, a 0.93 percentile increase in BMI, and a 0.48 mg/dL decrease in HDL-C concentrations.

## 4. Discussion

As has been reported elsewhere [7], we found a significant increase in the overall mean amount of SSB consumption over the past 16 years in these nationally representative samples of US adolescents aged 12–19 years. Specifically, compared to adolescents from the NHANES III study cohort, adolescents from the NHANES 1999–2004 study cohort consumed approximately 7% more SSB serving equivalents per day. However, our data suggest that this increase was primarily due to an increase in the amount of SSBs consumed by males in the high-SSB intake group alone. Furthermore, multivariate linear regression analyses showed that increased SSB consumption was independently associated with increased HOMA-IR, SBP, WC, and BMI percentile for age and sex and decreased HDL-C concentrations, reinforcing the negative impact that SSB consumption has on health parameters.

Given the strong association between SSB intake and altered metabolism [2, 25–29], our results are surprising. At the outset, we expected to find that SSB consumption had increased comparably among all consumption tiers of SSB intake and in both sexes, paralleling the increasing prevalence of obesity and MetS in the adolescent population [8–15]. However, our data suggest that the increase in SSB consumption that has been noted over the past 16 years in adolescents [7] is not universal, but rather confined to the top quintile of male SSB consumers, and that SSB consumption in most adolescents (the bottom four quintiles in males and all quintiles in females) has not increased over this time period. Thus, as has been reported elsewhere [45, 46], factors other than SSB consumption in the pediatric population—such as an increase in total caloric intake, a decrease in calcium and other nutrient intake, a decrease in daily physical activity, and potential genetic influences—must also be contributing to the increasing prevalence of pediatric obesity and MetS. 

Nevertheless, as shown by our linear regression analyses, SSB consumption is associated with adverse metabolic parameters [16–18]. It is thus reassuring that legislative and regulatory actions have specifically targeted SSB consumption in schools as one means to promote improved adolescent health [47]. Initiatives such as the one in 2006 between major US beverage manufacturers, the American Heart Association, and the Clinton Foundation to establish voluntary guidelines regarding the type of beverages sold at schools have brought national attention to the negative metabolic effects associated with SSB consumption, and although some reports suggest that initiatives to restrict SSB sales in schools may have only a marginal impact on overall SSB consumption [7] they are most likely at least in part responsible for the recent finding that the prevalence of high BMI among US children and adolescents between 2003-2004 and 2005-2006 has not changed [48]. Previous research has also shown a beneficial effect on body weight by reducing SSB consumption in those adolescents with an elevated BMI, supporting the current American Academy of Pediatrics guidelines to limit SSB consumption [49].

Moreover, our data show that SSB intake in the top quintile of SSB consumers from each of the NHANES 1999–2004 study periods has begun to decline (7.2 SSB serving equivalents per day in 1999-2000, to 7.1 SSB serving equivalents per day in 2001-2002, to 6.9 SSB serving equivalents per day in 2003-2004) after increasing dramatically from the amount of SSB consumption in NHANES III (6.5 SSB serving equivalents per day in 1988–1991, and 6.7 SSB serving equivalents per day in 1991–1994). Whether this trend continues, and whether it is associated with changes in the prevalence of pediatric obesity and MetS, warrants further investigation.

One limitation to this study is that the data are cross-sectional, and thus cannot infer causality; another is that the analyses are confined to adolescents aged 12–19 years. We can also not exclude the possibility that significant differences in either the age at which children begin consuming SSBs and/or the amounts of SSBs consumed among children in the pre-teen years increase the rate of obesity and MetS in adolescents, as both of these scenarios would not be captured by our analyses. Moreover, since the pubertal status of subjects participating in NHANES has not been documented since NHANES III, we are unable to longitudinally adjust for the subjects' degree of pubertal maturation. Studies such as ours that utilize questionnaires also have other inherent limitations: (i) the recall method is subject to inaccuracy and bias, especially with behaviors such as dietary habits [50] and levels of exercise [41], (ii) individual's dietary habits and levels of exercise can vary greatly from one day to the next, limiting the reliability of short-term recall on long-term patterns. However, given that overweight subjects often underreport their levels of energy intake [50] and less active adolescents often overestimate their degree of physical fitness [41], we can have confidence in our results since these biases would be expected to diminish rather than enhance our ability to find significant associations between SSB consumption and insulin resistance-associated measures.

## 5. Conclusions

Although the mean overall amount of SSB consumption in US adolescents has increased over the past 16 years, paralleling the increase in pediatric obesity and MetS, our data suggest that this observation is primarily due to increased SSB intake in the top quintile of male SSB consumers. Thus, increased consumption of SSBs is not solely responsible for the increasing prevalence of obesity and MetS for the majority of US adolescents. Public health efforts aimed at addressing other factors linked with adverse metabolic parameters and anthropometric measurements, such as decreasing physical activity and increasing consumption of fast foods and other calorie-dense and nutritionally-poor foods, are therefore needed to adequately address the ongoing epidemic of pediatric obesity and MetS.

## Figures and Tables

**Table 1:** Characteristics of US adolescents aged 12–19 years: NHANES III (1988–1994) and complete NHANES 1999–2004 cohorts.

	NHANES III	NHANES III					
	Phase I	Phase II	NHANES	NHANES	NHANES	NHANES III	NHANES
	1988–1991	1991–1994	1999-2000	2001-2002	2003-2004	1988–1994	1999–2004
Number of Participants (*n*)	1531	1703	2308	2417	2242	3234	6967
Age in years (mean)	15.7	15.2	15.4	15.5	15.4	15.4	15.5
Sex (%)							
Male	50.9	50.6	51.5	50.6	51.1	50.7	51.1
Female	49.1	49.4	48.5	49.4	48.9	49.3	48.9
Race/ethnicity (%)							
Non-Hispanic white	66.9	65.1	56.8	63.8	64.9	66.0	62.1
Non-Hispanic black	15.4	15.6	14.9	13.9	15.7	15.5	14.8
Mexican American	8.2	8.3	13.0	9.0	11.1	8.3	10.9
Other race–including multiracial	4.8	4.8	7.3	5.3	3.6	4.8	5.3
Other Hispanic	4.7	6.2	8.0	8.0	4.7	5.4	6.9

**Table 2:** Trends in SSB intake among adolescents aged 12–19 years: NHANES III (1988–1994), NHANES 1999-2000, NHANES 2001-2002, and NHANES 2003-2004. Low SSB intake defined as lowest quintile based on NHANES III cutoffs; medium defined as 2nd–4th quintiles; high defined as highest quintile. Mean indicates mean number of serving equivalents of sugar-sweetened beverages reported during 24 hour dietary recall. *Significant results are in bold*. Abbreviations: SSB, sugar-sweetened beverage; SE, standard error.

	NHANES III	NHANES III							
	Phase I	Phase II	NHANES	NHANES	NHANES		NHANES III	NHANES	
	1988–1991	1991–1994	1999-2000	2001-2002	2003-2004	Trends	1988–1994	1999–2004	
	Mean (SE)	Mean (SE)	Mean (SE)	Mean (SE)	Mean (SE)	*P* value^(a)^	Mean (SE)	Mean (SE)	*P* value^(b)^
SSB intake									
Overall	**2.6 (0.1)**	**3.0 (0.1)**	**3.2 (0.2)**	**2.9 (0.2)**	**3.0 (0.1)**	**.04**	**2.8 (0.1)**	**3.0 (0.1)**	**.04**
Low (0–0.7 serving equivalents)	0.04 (.01)	0.08 (0.03)	0.05 (0.01)	0.04 (0.01)	0.06 (0.01)	.96	0.06 (0.01)	.05 (0.01)	.52
Medium (0.8–4.5 serving equivalents)	2.5 (0.1)	2.4 (0.1)	2.5 (0.1)	2.4 (0.04)	2.6 (0.1)	.17	2.4 (0.1)	2.5 (0.03)	.12
High (4.6-24.2 serving equivalents)	6.5 (0.3)	6.7 (0.2)	7.2 (0.2)	7.1 (0.2)	6.9 (0.2)	.12	**6.6 (0.2)**	**7.1 (0.1)**	**.01**

^(a)^ *P* value for test for linear trend of sugar-sweetened beverage levels among NHANES III, NHANES 1999-2000, NHANES 2001-2002, NHANES 2003-2004. ^(b)^ *P* value comparing NHANES III to NHANES 1999–2004

**Table 3:** Trends in SSB Intake among adolescents aged 12–19 years by sex: NHANES III (1988–1994), NHANES 1999-2000, NHANES 2001-2002, and NHANES 2003-2004. Low SSB intake defined as lowest quintile based on NHANES III cutoffs; medium defined as 2nd–4th quintiles; high defined as highest quintile. Mean indicates mean number of serving equivalents of sugar-sweetened beverages reported during 24 hour dietary recall. *Significant results are in bold*. Abbreviations: SSB, sugar-sweetened beverage; SE, standard error.

	NHANES III	NHANES III							
	Phase I	Phase II	NHANES	NHANES	NHANES		NHANES III	NHANES	
	1988–1991	1991–1994	1999-2000	2001-2002	2003-2004	Trends	1988–1994	1999–2004	
	Mean (SE)	Mean (SE)	Mean (SE)	Mean (SE)	Mean (SE)	*P* value^(a)^	Mean (SE)	Mean (SE)	*P* value^(b)^
**Males**									
SSB intake									
Overall	**3.1 (0.1)**	**3.5 (0.2)**	**3.8 (0.2)**	**3.5 (0.3)**	**3.6 (0.1)**	**.048**	3.3 (0.1)	3.6 (0.1)	. 51
Low (0-0.7 serving equivalents)	0.02 (0.01)	0.09 (0.06)	.03 (0.01)	0.04 (0.01)	0.05 (0.02)	.99	0.05 (0.03)	0.04 (0.01)	.57
Medium (0.8-4.5 serving equivalents)	2.6 (0.1)	2.4 (0.1)	2.6 (0.1)	2.6 (0.04)	2.7 (0.1)	.18	2.5 (0.1)	2.6 (0.04)	.08
High (4.6-24.2 serving equivalents)	6.6 (0.2)	6.9 (0.3)	7.5 (0.3)	7.3 (0.3)	7.2 (0.2)	.08	**6.8 (0.2)**	**7.3 (0.2)**	**.03 ** ^?>^

**Females**									
SSB intake									
Overall	2.1 (0.2)	2.5 (0.1)	2.5 (0.2)	2.3 (0.1)	2.4 (0.1)	.35	2.3 (0.1)	2.5 (0.1)	.30
Low (0–0.7 serving equivalents)	0.05 (0.01)	0.08 (0.03)	0.07 (0.01)	0.05 (0.01)	0.06 (0.01)	.92	0.06 (0.02)	0.06 (0.01)	.93
Medium (0.8–4.5 serving equivalents)	2.4 (0.1)	2.4 (0.1)	2.5 (0.04)	2.3 (0.1)	2.5 (0.1)	.44	2.4 (0.1)	2.4 (0.03)	.47
High (4.6–24.2 serving equivalents)	6.1 (0.5)	6.2 (0.3)	6.6 (0.3)	6.7 (0.2)	6.3 (0.3)	.52	6.2 (0.3)	6.5 (0.1)	.26

^(a)^ *P* value for test for linear trend of sugar-sweetened beverage levels among NHANES III, NHANES 1999-2000, NHANES 2001-2002, NHANES 2003-2004. ^(b)^ *P* value comparing NHANES III to NHANES 1999–2004

**Table 4:** Regression analyses of metabolic parameters and anthropometric Measurements associated with SSB intake among US adolescents aged 12–19 years: NHANES III (1988–1994), NHANES 1999-2000, NHANES 2001-2002, NHANES 2003-2004, and complete NHANES 1999–2004 cohorts. Beta (SE) represents the number of SSBs consumed per day, adjusting for amount of physical activity performed per day, age, sex, ethnicity, and energy intake per day (in kilocalories). Abbreviations: HOMA-IR, homeostasis model assessment of insulin resistance; TC, total cholesterol; TG, triglyceride; HDL-C, high-density lipoprotein cholesterol; LDL-C, low-density lipoprotein cholesterol; SBP, systolic blood pressure; DBP, diastolic blood pressure; BMI, body mass index; WC, waist circumference; SE, standard error.

	NHANES III	NHANES III					
	Phase I	Phase II	NHANES	NHANES	NHANES	NHANES III	NHANES
	1988–1991	1991–1994	1999-2000	2001-2002	2003-2004	1988–1994	1999–2004
	Beta (SE)	Beta (SE)	Beta (SE)	Beta (SE)	Beta (SE)	Beta (SE)	Beta (SE)
HOMA-IR^#^	—	—	0.08 (0.04)	0.03 (0.02)	**0.06 (0.02)∗**	—	0.06(0.02)*
TC (mg/dL)	−0.51 (0.55)	−0.53 (0.47)	−0.56 (0.41)	0.04 (.30)	0.17 (0.50)	−0.52 (.37)	−0.11 (.23)
HDL-C (mg/dL)	−0.80(0.22)*	−0.16 (0.15)	0.64(0.17)*	−0.27(0.12)*	−0.53(0.11)*	−0.42(0.12)*	−0.48(0.08)*
LDL-C (mg/dL)^#^	0.93 (0.79)	0.70 (0.71)	−0.27 (0.48)	−0.14 (0.54)	0.79 (0.39)	0.72 (0.51)	0.13 (0.29)
TG (mg/dL)^#^	−0.33 (1.58)	−2.58 (1.87)	1.41 (1.15)	0.04 (1.87)	1.53 (1.14)	−1.29 (1.18)	1.02 (0.84)
TG/HDL-C ratio^#^	0.02 (0.04)	−0.03 (0.05)	0.05 (0.04)	−0.01 (0.05)	0.05 (0.03)	0.001 (0.03)	0.03 (0.02)
SBP (mmHg)	0.08 (0.16)	0.12 (0.17)	0.11 (0.12)	0.20(0.10)*	0.15 (0.17)	0.10 (0.14)	0.16(0.07)*
DBP (mmHg)	−0.05 (0.16)	−0.17 (0.20)	−0.09 (0.11)	−0.04 (0.08)	0.13 (0.16)	−0.10 (0.16)	0.01 (0.07)
WC (cm)	0.42 (0.23)	0.07 (0.25)	0.40(0.19)*	0.47(0.15)*	0.54(0.10)*	0.22 (0.18)	0.47(0.08)*
BMI (kg/m^2^) percentile for age-sex	0.70 (0.42)	0.04 (0.67)	0.66(0.29)*	0.93(0.37)*	1.13(0.25)*	0.38 (0.45)	0.93(0.18)*

^#^morning & fasting subsample.  *P < .05 (**Significant results are in bold**).
